# Kinetic Determination of Ribavirin in Drug Formulations

**Published:** 2007-03

**Authors:** A. M. El-Brashy, Z. A. Sheribah, M. K. Sharaf El-Din, R. M. El-Gamal

**Affiliations:** *Department of Analytical Chemistry, Faculty of Pharmacy, University of Mansoura, Mansoura, Egypt*

**Keywords:** spectrophotometry, ribavirin, potassium permanganate, dosage forms

## Abstract

Two simple and sensitive kinetic methods were developed for the determination of ribavirin in bulk and in its pharmaceutical preparations using alkaline potassium permanganate as an oxidizing agent. The methods are based upon a kinetic investigation of the oxidation reaction of the drug at room temperature for fixed times of 20 and 30 minutes. In the first method, the absorbance of the colored manganate ion was measured at 610 nm, while in second method the reduction in the absorbance of permanganate was measured at 525 nm. The absorbance concentration plots were linear over the range of 3-15 μg/ml with detection limits of 0.028 μg/ml in the first method and 0.229 μg/ml for the second method. The proposed methods were applied successfully for the determination of the drug in its pharmaceutical formulations, the percentage recoveries were 100.15 ± 1.34, 100.06 ± 0.86 in the first method, and 99.60 ± 0.54, 100.43 ± 0.82 in the second method. The results obtained were compared statistically with those obtained by the official method and showed no significant differences regarding accuracy and precision.

## INTRODUCTION

**Figure 1:**
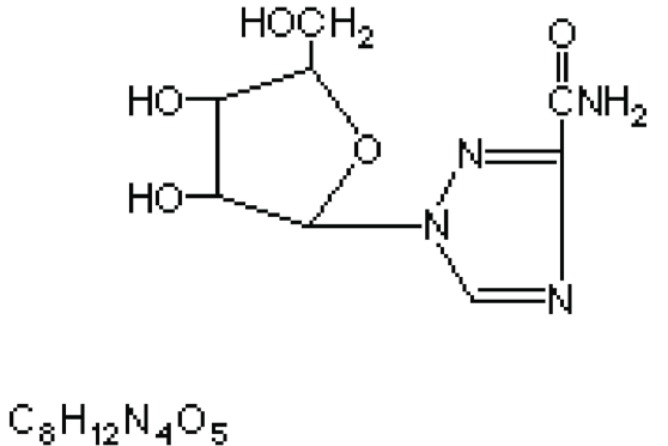
Structure of Ribavirin.

Ribavirin (1-beta-D-ribofuranosyl-1H-1, 2, 4 thiazole-3-carboxamine) (Fig. 1) is a purine nucleoside analog with a modified base and D- ribose sugar (1). It inhibits the replication of a wide range of RNA and DNA viruses, including orthomyxo-, paramyxo-, arena-, bunya-, herpes-, adeno-, pox- and retro viruses. *In vitro* inhibitory concentration range is 3-10 μg/ml for influenza, parainfluenza and respiratory syncytical (RSV) viruses (2). Similar concentrations may reversibly inhibit macromolecular synthesis and proliferation of uninfected cells and suppress lymphocytes responses *in vitro*. The reported methods for the determination of the drug include fluorimetry (3) spectrophotometry (4-6) and high performance liquid chromatography (HPLC) (7-10).

The catalytic kinetic spectrophotometric method is one of the most attractive approaches for the ultratrace determination of certain chemicals and has many advantages:

- Selectivity due to the measurement of the evolution of the absorbance with time of reaction instead of the measure of concrete absorbance value;
- Possibility of no interference of the colored and of turbidity background of the sample;
- Possibility of no interference of other active compounds present in the commercial products, if they are resisting the chemical reaction conditions established for the proposed kinetic method (11).

The aim of the present work was to study the reaction between ribavirin and potassium permanganate in alkaline medium kinetically by two different methods in an attempt to evaluate the drug in its dosage forms. The proposed spectrophotometric methods were simple and did not need sophisticated instruments or special skills, sensitive, rapid and readily adaptable to both the bulk drug and dosage forms.

## EXPERIMENTAL

### Apparatus

UV - 1601, Shimadzu recording spectrophotometer (P/N 206 - 67001) equipped with kinetic accessory provided with temperature controlled cell (TCC - 240A) thermoelectrical temperature. Recording range, 0-1; wave-length, 610 and 525 nm; factor 1; number of cell, 1; reaction times, 20 and 30 min and cycle time, 0.1 min.

### Materials and Reagents

- Ribavirin was kindly obtained from T3A (Cairo, Egypt). The purity of the drug was determined and confirmed by applying the official method (12).
- Pharmaceutical preparations containing the drug were purchased from different commercial sources in the local markets. Ribavirin 200 capsules: labeled to contain 200 mg ribavirin/capsule (lot No. 020303; T3A, Cairo, Egypt); Viracure capsules: labeled to contain 200 mg ribavirin/capsule (lot No. B31120; October Pharma Co, Cairo, Egypt).
- Reagents: All the reagents used were of analytical grade and water was always double distilled. Aqueous solutions of 7.59 × 10^-2^, 7.59 × 10^-3^ M potassium permanganate (Merck, Germany) and 2 M NaOH (BDH, UK) were prepared.
- Stock solutions.

The stock solution of the studied drug was prepared by dissolving 100 mg of ribavirin in 100 ml distilled water and solicited for few minutes. Working standard solutions were prepared by dilution of the stock solution with the same solvent. The solutions were stable for one week if kept in the refrigerator.

### General procedures

**Construction of the calibration graph for the first method.** An aliquot solutions of ribavirin containing 30-150 μg was transferred into a 10 ml volumetric flask, 2.5 ml of 2 M NaOH was added followed by 0.7 ml of 7.59 × 10^-2^ M KMnO_4_, the mixture was shaken well and completed to the volume with distilled water. The absorbance was scanned during 20 min. at room temperature at 610 nm against a similar blank prepared simultaneously.

**Construction of the calibration graph for the second method.** An aliquot solution of ribavirin containing 30-150 μg was transferred into a 10 ml volumetric flask, 3ml of 2 M NaOH was added followed by 1 ml of 7.59 × 10^-3^ M KMnO_4_, the mixture was shaken well and completed to the volume with distilled water. The reduction in absorbance was scanned during 30 min. at room temperature at 610 nm against a similar blank prepared simultaneously.

**Procedures for determination of ribavirin in its dosage forms.** An accurately weighed quantity of the mixed contents of 10 capsules equivalent to 50 mg of the drug was transferred into a 100 ml volumetric flask. About 70 ml distilled water were added and the mixture was sonicated for 15 min, filtered and then diluted to volume with distilled water. An aliquot of the filtrate was transferred into a 10 ml volumetric flask and either above procedure was adopted. The nominal content of the capsules were calculated by referring to the prepared calibration graphs or the corresponding regression equations.

## RESULTS AND DISCUSSION

The reaction between ribavirin and KMnO_4_ in alkaline medium yields a green color due to the production of manganate ions, which absorb at 610 nm. As the intensity of the color increases with time, this was used as a useful method for the determination of ribavirin in bulk as well as in dosage forms (first method).

At the same time owing to the consumption of KMnO_4_ in the reaction the absorbance of KMnO_4_ peaking at 525 nm decreases with time. This was also used as a useful method for the determination of ribavirin (second method).

The various experimental parameters affecting the development and stability of the reaction product in either method were optimized by changing each variable in turn while keeping all others constant.

### Effect of KMnO_4_

**Figure 2:**
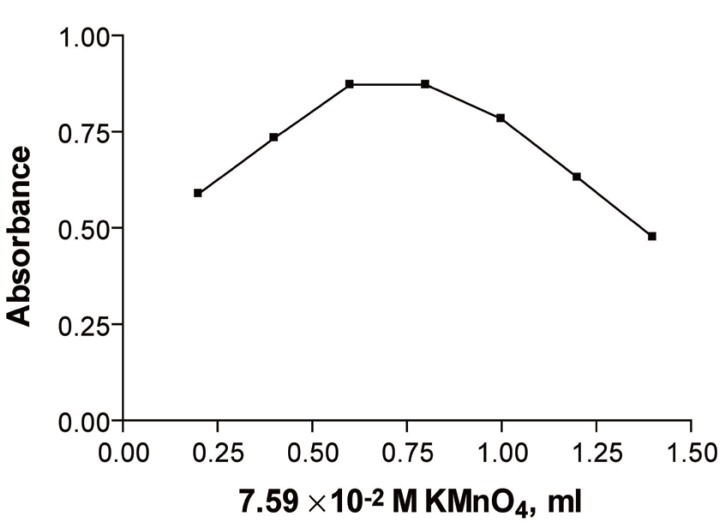
Effect of volume of potassium permanganate on the reaction product of 6.143 × 10^-5^ M ribavirin measured at room temperature after 20 min. (Method A).

In the first method, the reaction rate and maximum absorbance increased with increasing KMnO_4_ concentration. It was found that 0.6 ml of 7.59 × 10^-2^ M KMnO_4_ was adequate for the maximum absorbance. Higher concentrations of KMnO_4_ yielded lower absorbance values probably due to decomposition of the product (Fig. 2).

**Figure 3:**
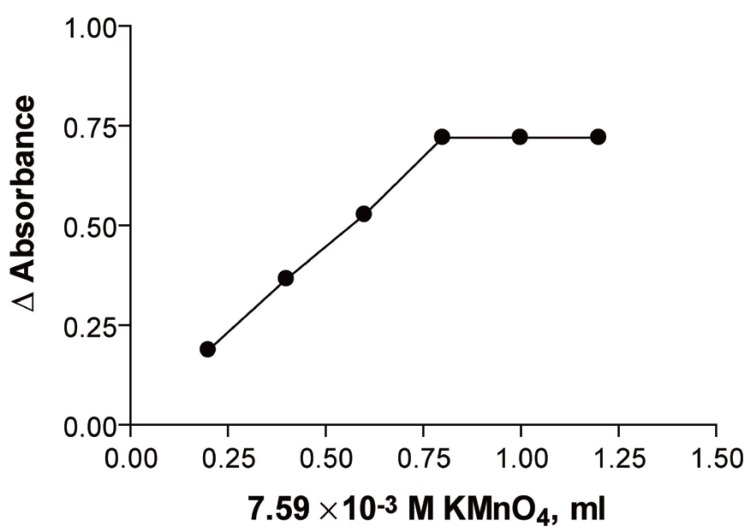
Effect of volume of sodium hydroxide on the reaction product of 6.143 × 10^-5^ M ribavirin measured at room temperature after 20 min. (Method A).

While in the second method, the reaction rate and maximum absorbance reduction increased with increasing KMnO_4_ concentration. It was found that 1 ml of 7.59 × 10^-3^ M KMnO_4_ was adequate for the maximum absorbance reduction (Fig. 3).

### Effect of NaOH

**Figure 4:**
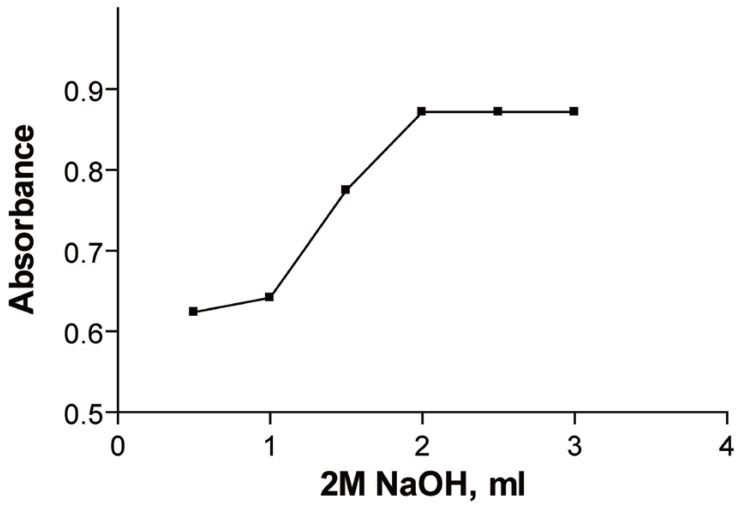
Effect of volume of potassium permanganate on the reaction product of 6.143 × 10^-5^ M ribavirin measured at room temperature after 30 min. (Method B).

It was found that increasing the volume of 2 M NaOH would increase the absorbance of the reaction product up to 2.5 ml. (In the first method) (Fig. 4).

**Figure 5:**
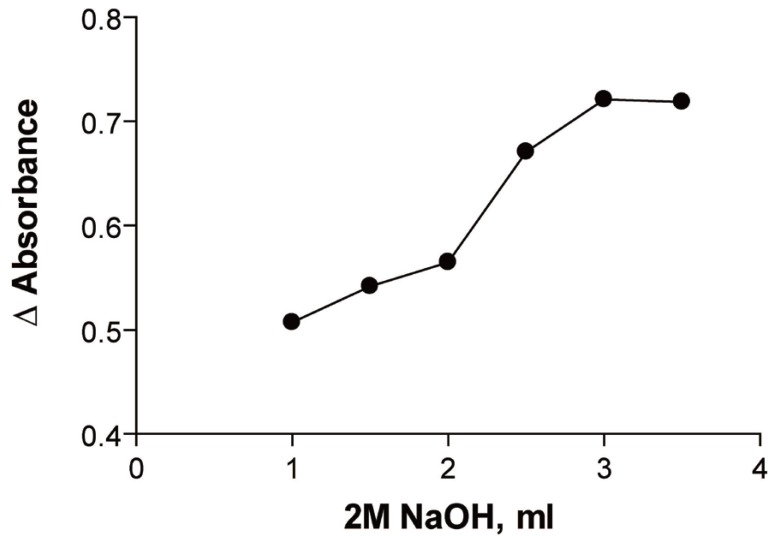
Effect of volume of sodium hydroxide on the reaction product of 6.143 × 10^-5^ M ribavirin measured at room temperature after 30 min. (Method B).

In the second method increasing the volume of 2 M NaOH would increase the reduction in the absorbance of KMnO_4_ up to 3 ml (Fig. 5).

The rate of the reaction was found to be dependent on ribavirin concentration. The rate was followed at room temperature with various concentrations in the range of 3-5 μg/ml keeping KMnO_4_ and NaOH concentrations constant.

The reaction rate was found to obey the following equation:

(Eq. 1) $$\mathrm{Rate}=K'{\left[\mathrm{drug}\right]}^{n}$$

where K` is the pseudo - order rate constant and n is the order of the reaction.

The rate of the reaction in either method may be estimated by the variable time method measurement as ΔA/Δt, where A is the absorbance and t is the time in seconds. Taking logarithms of rate and concentrations (Table 1), Eq.1 is transformed into

(Eq. 2) Log rate=logΔA/Δt=log K'+n logdrug

**Table 1:** Logarithms of rate for different concentrations of ribavirin at room temperature and at 610 nm, 525 nm

At 610 nm		At 525 nm	
Log ΔA/Δt	Log [Ribavirin] (M)	Log ΔA/Δt	Log [Ribavirin] (M)

-3.844	-4.910	-4.059	-4.910
-3.608	-4.609	-3.859	-4.689
-3.445	-4.433	-3.567	-4.388
-3.335	-4.309	-3.529	-4.309
-3.285	-4.212	-3.436	-4.212

Log (rate) versus log [drug] gave the regression equation:

Log rate=0.2049+0.825 log C  r=0.9986 in the first method

Hence K` = 1.603 S^-1^ and the reaction is first order (n = 0.825).

Log rate=0.3442+0.896 log C  r=0.9988 in the second method

Hence K` = 2.209 S^-1^ and the reaction is first order (n=0.896).

### Evaluation of the kinetic methods

The quantitation of the drug under the optimized experimental conditions outlined above would result in a pseudo - first order with respect to the drug concentration where KMnO_4_ concentration was at least 74 times of the initial concentration of the drug in the first method or 12 times of the initial concentration of the drug in the second method.

However the rate will be directly proportional to drug concentration in a pseudo - first rate equation as follows:

(Eq. 3) $$\mathrm{Rate}=K'\left[\mathrm{drug}\right]$$

where K` is the pseudo order rate constant.

Several experiments were then carried out to obtain drug concentration from the rate data according to (Eq. 3). Initial rate, rate constant, fixed concentration and fixed time methods (14, 15), were tried and the most suitable analytical method was selected taking into account the applicability, the sensitivity, the intercept and the correlation coefficient (r).

### Rate-constant method

Graphs of log absorbance versus time for ribavirin in the range of 1.229 × 10^-5^ - 6.143 × 10^-5^ M were plotted and all appeared to be rectilinear. Pseudo - first order rate constant (K`) corresponding to different drug concentrations (C) were calculated from the slope multiplied by -2.303 and are presented in Table 2.

**Table 2:** Values of K` calculated from slopes of log A versus time graphs at 610 nm and 525 nm

At 610 nm		At 525 nm	
K` (S^-1^)	[Ribavirin] (M)	K` (S^-1^)	[Ribavirin] (M)

-5.773 × 10^-4^	1.229 × 10^-5^	-5.601 × 10^-4^	1.229 × 10^-5^
-4.368 × 10^-4^	2.457 × 10^-5^	-7.188 × 10^-4^	2.048 × 10^-5^
-4.806 × 10^-4^	3.686 × 10^-5^	-6.453 × 10^-4^	4.095 × 10^-5^
-4.184 × 10^-4^	4.914 × 10^-5^	-5.354 × 10^-4^	4.914 × 10^-5^
-3.293 × 10^-4^	6.143 × 10^-5^	-5.846 × 10^-4^	6.143 × 10^-5^

Regression of (C) versus K` gave equations:

K'=−6.028×10−4+4.187 C  r=0.897 in the first method

K'=−6.877×10−4+2.683 C  r=0.585 in the second method

### Fixed-concentration method

Reaction rates were recorded for different concentrations of the drug in the range of 2.457 × 10^-5^ - 4.914 × 10^-5^ M in the first method and 1.229 × 10^-5^ - 6.143 × 10^-5^ in the second method. Preselected values of the absorbance (0.3) in the first method and (1.1) in the second method were fixed and the time was measured in seconds. The reciprocal of times (1/t) versus the initial concentrations of drug (Table 3) were plotted and the following equations of the calibration graphs were obtained:

1/t=−8.363×10−3+386.350 C  r=0.9845 in the first method

1/t=−5.989×10−4+94.028 C  r=0.9814 in the second method

**Table 3:** Values of reciprocal of time taken at fixed absorbance for different rates of variable concentrations of ribavirin at constant concentrations of NaOH and KMnO_4_ at room temperature

At 610 nm		At 525 nm	
1/t (S^-1^)	[Ribavirin] (M)	1/t (S^-1^)	[Ribavirin] (M)

1.618 × 10^-3^	2.457 × 10^-5^	8.547 × 10^-4^	1.229 × 10^-5^
4.902 × 10^-3^	3.686 × 10^-5^	1.267 × 10^-3^	2.048 × 10^-5^
11.111 × 10^-3^	4.914 × 10^-5^	2.688 × 10^-3^	4.095 × 10^-5^
		3.968 × 10^-3^	4.914 × 10^-5^
		5.556 × 10^-3^	6.143 × 10^-5^

### Fixed-time method

Reaction rates were determined for different concentrations of the drug. At a preselected fixed time, which was accurately determined, the absorbance was measured. Calibration graphs of absorbance versus initial concentrations of ribavirin were established at fixed times of 5, 10, 15, 20 min. in the first method and 5, 10, 15, 20, 25, 30 min. in the second method with the regression equations assembled in (Table 4).

**Table 4:** Regression equation for ribavirn at different fixed time over the range of 1.229 × 10^-5^ to 6.143 × 10^-5^

At 610 nm			At 525 nm		
Time (min)	Regression equation	(r)^a^	Time (min)	Regression equation	(r)^a^

5	A= -0.0335 + 0.0438 C	0.9967	5	A= -0.0221+ 0.0222 C	0.8943
10	A= -0.0114 + 0.0516 C	0.9992	10	A= -0.0207+ 0.0327 C	0.9951
15	A= 1.559 × 10^-3^ + 0.0557 C	0.9999	15	A= -0.0166 + 0.0391 C	0.9979
20	A= 0.0101 + 0.575 C	0.9999	20	A= -7.810 ×10^-3^ + 0.0431 C	0.9994
			25	A= 3.382 ×10^-3^ + 0.0456 C	0.9999
			30	A= 0.0163 + 0.0469 C	0.9999

a Correlation coefficient.

It is clear that the slope increased with time and the most acceptable values of the correlation coefficient (r) and the intercept were chosen as the most suitable time interval for measurement.

### Calibration graphs

After optimizing the reaction conditions, the fixed time was applied to the determination of the drug in pure form over the concentration range 3-5 μg/ml. Analysis of the data gave the following regression equations:

A=0.0101+0.0575 C  r=0.9999 in the first method

A=0.0163+0.0469 C  r=0.9999 in the second method

**Figure 6:**
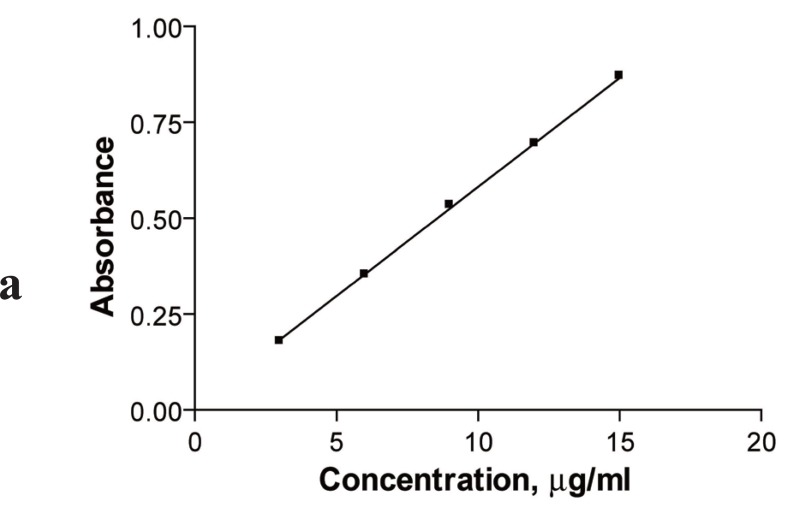
Kinetic spectrophotometric calibration curve for the reaction between ribavirin and alkaline potassium permanganate. (Method A).

**Figure 7:**
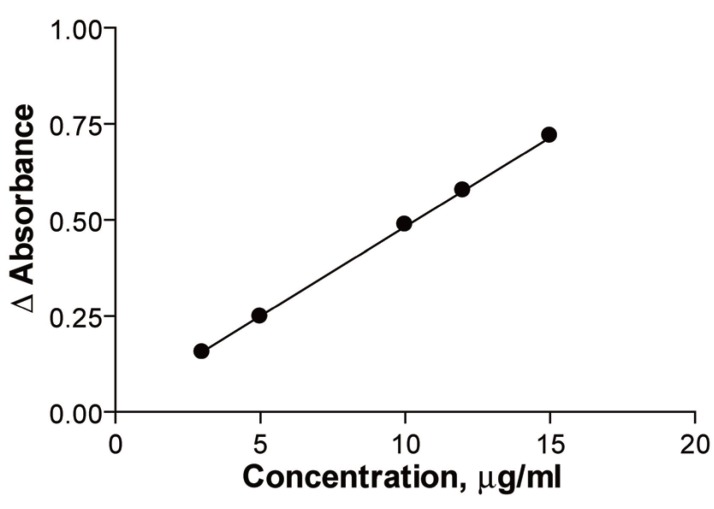
Kinetic spectrophotometric calibration curve for the reaction between ribavirin and alkaline potassium permanganate. (Method B).

The calibration graphs were shown in (Figs. 6, 7), the % recoveries of the drug compared with that obtained by the official method (12), were given in (Table 5).

**Table 5:** Validity of the proposed method for the determination of the studied drug

Proposed methods						Official method		
1^st^ method			2^nd^ method					
Amount taken μg/ml	Amount found μg/ml	Recovery %	Amount taken μg/ml	Amount found μg/ml	Recovery %	Amount taken μg/ml	Amount found μg/ml	Recovery %

3	2.955	98.49	3	3.000	100	5	4.913	98.26
6	5.998	99.97	5	4.983	99.66	10	10.064	100.64
9	9.129	101.43	10	10.079	100.79	15	15.028	100.19
12	11.929	99.41	12	11.977	99.81	20	20.100	100.50
15	14.990	99.93	15	15.004	100.03	25	24.895	99.58
X^a^		99.85			100.06			99.83
SD		1.07			0.44			0.97
t		0.03 (2.31)^a^			1.008 (2.31)^a^			
F		1.21 (6.39)^a^			4.95 (6.39)^a^			

Each result is the average of three separate determinations.

a The values between brackets are the tabulated student t-test and variance ratio test (at *P*=0.05) (16). X, mean; SD, Standard deviation.

Statistical analysis (16) of the results obtained by the proposed and reference method (13) using student’s t test and variance ratio revealed no significant difference between the performance of the methods regarding accuracy and precision.

The proposed methods were successfully applied for determination of the studied drug in its dosage forms, as shown in (Table 6), compared with the result obtained by the reference method.

**Table 6:** Application of the proposed methods to the determination of the studied drug in dosage forms

Preparation	1^st^ method			2^nd^ method			Official method		
	Amount taken μg/ml	Amount found μg/ml	Recovery %	Amount taken μg/ml	Amount found μg/ml	Recovery %	Amount taken μg/ml	Amount found μg/ml	Recovery %

Ribavirin 200 capsules (ribavirin, 200mg/capsule)	3	3.017	100.55	3	3.008	100.27	5	4.943	98.85
	9	8.916	99.07	5	5.019	100.39	10	9.946	99.46
	12	12.068	100.57	10	10.178	101.78	15	15.072	100.48
				12	12.016	100.13			
				15	14.934	99.56			
Mean ± SD			100.06 ± 0.86			100.43 ± 0.82			99.29 ± 0.81
Student’s t test			1.07 (2.78)^a^			1.84 (2.45)^a^			
F test			1.13 (19)^a^			1.03 (19.25)^a^			
Viracure 200 capsules (ribavirin, 200mg/capsule)	3	3.050	101.67	3	2.997	99.09	5	4.961	99.22
	6	5.949	99.16	5	5.005	100.09	10	10.156	101.56
	9	8.966	99.63	10	9.929	99.29	15	15.101	100.67
				12	12.032	100.27			
				15	14.891	99.27			
Mean ± SD			100.15±1.34			99.60±0.54			100.48 ± 1.18
Student’s t test			-0.32 (2.78)^a^			-1.504(2.45)^a^			
F test			1.28 (19)^a^			4.85 (6.94)^a^			

Each result is the average of three separate determinations.

a The values between brackets are the tabulated student t-test and variance ratio test (at *P*=0.05) (16).

### Mechanism of the reaction

**Figure 8:**
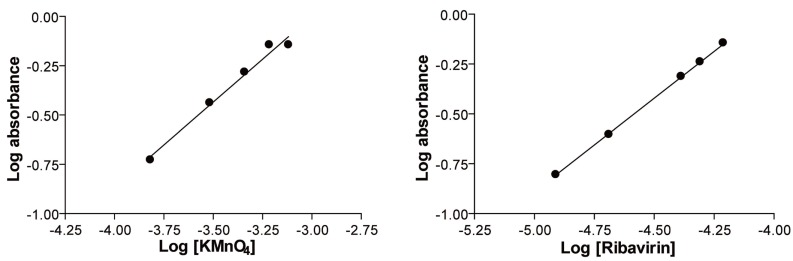
Stoichiometry of the reaction between ribavirin and alkaline potassium permanganate adopting limiting logarithmic method.

**Figure 9:**
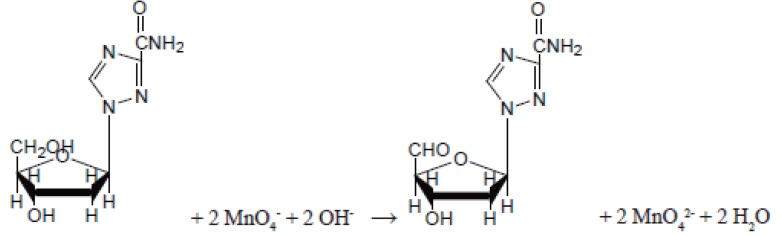
The proposed pathway of the reaction.

The stoichiometry of the reaction was studied adopting the limiting logarithmic method (17). The ratio of the reaction between ribavirin and KMnO_4_ in alkaline medium was calculated by dividing the slope of KMnO_4_ curve over the slope of the drug curve (Fig. 8a, 8b). It was found that the ratio was (1:1) KMnO_4_ to drug. The proposed pathway of the reaction is given in Figure 9.

## CONCLUSION

The proposed methods were simple, accurate, precise, sensitive, rapid and low cost. Furthermore, the proposed methods do not require elaboration of procedures, which are usually associated with chromatographic methods. The proposed methods could be applied successfully for determination of the studied drug in pure form as well as in dosage form.
